# A year in the life of a North Atlantic seabird: behavioural and energetic adjustments during the annual cycle

**DOI:** 10.1038/s41598-020-62842-x

**Published:** 2020-04-07

**Authors:** Ruth E. Dunn, Sarah Wanless, Francis Daunt, Michael P. Harris, Jonathan A. Green

**Affiliations:** 1 School of Environmental Sciences, University of Liverpool, Liverpool, UK; 2 UK Centre for Ecology and Hydrology, Bush Estate Penicuik, UK

**Keywords:** Ecology, Animal migration, Behavioural ecology, Ecophysiology

## Abstract

During their annual cycles, animals face a series of energetic challenges as they prioritise different life history events by engaging in temporally and potentially spatially segregated reproductive and non-breeding periods. Investigating behaviour and energy use across these periods is fundamental to understanding how animals survive the changing conditions associated with annual cycles. We estimated year-round activity budgets, energy expenditure, location, colony attendance and foraging behaviour for surviving individuals from a population of common guillemots *Uria aalge*. Despite the potential constraints of reduced day lengths and sea surface temperatures in winter, guillemots managed their energy expenditure throughout the year. Values were high prior to and during the breeding season, driven by a combination of high thermoregulatory costs, diving activity, colony attendance and associated flight. Guillemots also exhibited partial colony attendance outside the breeding season, likely supported by local resources. Additionally, there was a mismatch in the timing of peaks in dive effort and a peak in nocturnal foraging activity, indicating that guillemots adapted their foraging behaviour to the availability of prey rather than daylight. Our study identifies adaptations in foraging behaviour and flexibility in activity budgets as mechanisms that enable guillemots to manage their energy expenditure and survive the annual cycle.

## Introduction

The annual cycles of seasonally breeding organisms are composed of life history events such as reproduction, post-breeding recovery, moult, migration, wintering, and preparation for the following breeding period^1^. A multitude of ecological and physiological processes, that vary temporally, underpin these organismal annual cycles. For example, animals experience seasonally-driven fluctuations in resource availability and environmental conditions^2^. To survive their annual cycles when faced with varying environmental conditions, animals must adjust their behaviour and balance their energy acquisition and expenditure^3^.

The reproductive season has previously been highlighted as an energetically costly period in an animal’s annual cycle^4,5^. High values of energy expenditure during the breeding period are incurred by parents making physiological and behavioural adjustments in order to invest in reproduction, through activities such as egg production and offspring provisioning, whilst also maintaining their own body condition at a level that safeguards future survival and breeding^6,7^. For example, in species that adopt central-place foraging strategies during reproduction, high energetic costs are often driven by increased allocation of time to energetically expensive commuting behaviours^8^. Outside the breeding season many taxa adopt costly migratory strategies as an adaptive behavioural response to seasonal variation in environmental conditions, resource availability and subsequent energy intake^9,10^. Animals may travel large distances to avoid energetically challenging areas and instead maximise energetic inputs, on the basis that the non-breeding location is sufficiently profitable to offset the costs of migration^11^. Thus, an organism’s movement, behaviour and external environment are all key factors in its energy expenditure during the annual cycle.

In addition to variation in migration strategies, many animals exhibit behavioural plasticity at hourly or daily scales in order to respond to variation in their environmental conditions. For example, basking sharks *Cetorhinus maximus* adopt a habitat-specific foraging strategy that is consistent with the daily vertical movements of their prey, to maximise energy intake versus expenditure^12^. Additionally, animals might display differences in their behavioural budgets associated with their state or age, for example if one sex allocates more time to foraging or risk-averse behaviours due to sex differences in the time and energy required for parental care^13^. However, despite evidence of behavioural plasticity across numerous taxa, the energetic consequences of such plasticity over the annual cycle have rarely been assessed.

In this study, we present the first estimates of year-round daily energy expenditure (DEE) for surviving individuals in a population of free-ranging common guillemots (hereafter ‘guillemots’) *Uria aalge* and seek to understand the behavioural and energetic adaptations that they utilised in order to survive the annual cycle. Guillemots have the highest wing loading (mass per unit area of the wing) of any flying bird^14^ and are central place foragers during their summer breeding seasons^15^. They therefore incur high energetic costs during the breeding period^16^, although these costs must remain below an optimal sustainable threshold (4 to 5 times their basal metabolic rate (BMR)^6,17^) to ensure reproductive success and survival. Following the costly breeding season, guillemots moult and then spend the winter primarily at sea, although some populations also exhibit non-breeding colony attendance^18,19^. Previous studies have shown that guillemots exhibit behavioural plasticity in response to variation in the environmental conditions they encounter during the non-breeding period, with consequences for their energetic budgets and mortality. For example, those that wintered in the Norwegian, Barents and White Seas increased their foraging effort ahead of several weeks of polar night, potentially maximising prey intake prior to this period of intense environmental constraint^20^. Furthermore, guillemots that over-wintered on the Newfoundland Shelf increased their diurnal foraging effort in response to seasonally varying vertical distributions of prey resources^21^. Thus guillemots, as with other seasonally breeding diving birds, make good models to investigate behavioural and energetic responses to seasonally varying ecological drivers.

We investigated the year-round behavioural and energetic adjustments made by a temperate population of breeding guillemots that may face similar constraints to more northerly populations, but which also adopt a strategy of returning to their breeding colony outside the breeding season. Our data span a year that was marked by low survival and subsequent breeding success^22^, hence increasing its optimality to investigate the strategies that surviving individuals exhibited. Therefore, within this study we investigated three questions. Firstly, how did the energy expenditure of surviving adult guillemots vary throughout the annual cycle? Secondly, how did these guillemots adjust their activity budgets and overwinter behaviour, in terms of migration and periodic returns to the colony, in order to balance their energy expenditure under varying environmental conditions? Thirdly, did guillemots adjust their diurnal diving behaviour in response to changes in daylight availability over the annual cycle?

## Results

**Figure 1:**
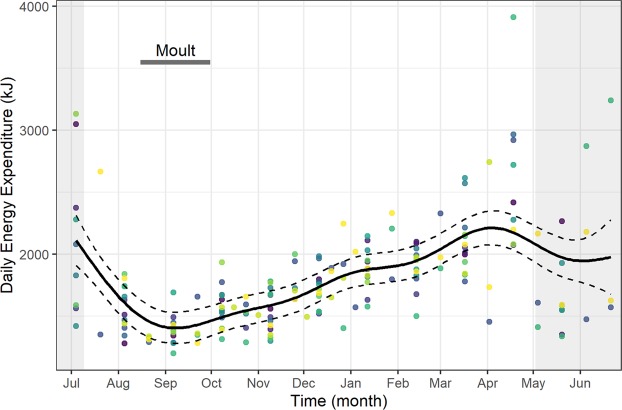
Annual daily energy expenditure of common guillemots. The daily energy expenditure of common guillemots from the Isle of May throughout the annual cycle (4^th^ July 2005–21^st^ June 2006). Mean estimated smoothing function (solid line) with upper and lower confidence intervals at two standard errors above and below the mean (dashed lines) from generalized additive mixed models. We also present the raw data points, coloured by individual. The breeding season is shaded in grey and the horizontal dark bar corresponds to the moult period.

Daily Energy Expenditure (DEE) of guillemots showed clear temporal changes over the annual cycle but with no substantial effect of sex (Supplementary Table S1). Values were relatively high at the end of the breeding season in July, but decreased markedly in August and September before showing a gradual and sustained increase over the non-breeding period (Fig. 1). DEE was lowest in September (generalised additive mixed model (GAMM) prediction = 1404 kJ) and highest in April (GAMM prediction = 2212 kJ).

**Figure 2:**
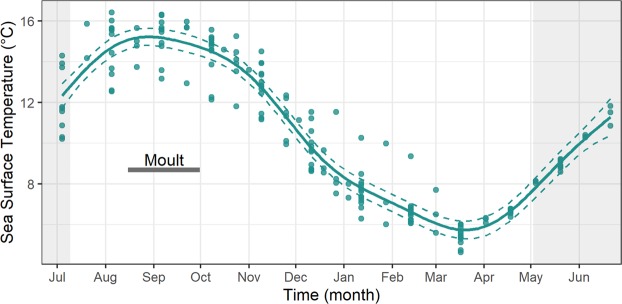
Annual sea surface temperature. Logger-derived sea surface temperature (SST) throughout the annual cycle (4^th^ July 2005–21^st^ June 2006). Mean estimated smoothing function (solid line) with upper and lower confidence intervals at two standard errors above and below the mean (dashed lines) from generalized additive mixed models. We also present the raw data points. The breeding season is shaded in light grey and the horizontal dark bar corresponds to the moult period.

Thermal conditions, as indicated by sea surface temperature (SST), experienced by individual guillemots varied across the annual cycle (Supplementary Table S1). Low values of DEE in August and September corresponded to high values of SST during the same months (14.9 ± 0.3 ^o^C and 15.2 ± 0.3 ^o^C respectively; Fig. 2) while the April peak in DEE occurred shortly after minimum SST in March (5.7 ± 0.2 ^o^C; Fig. 2).

**Figure 3:**
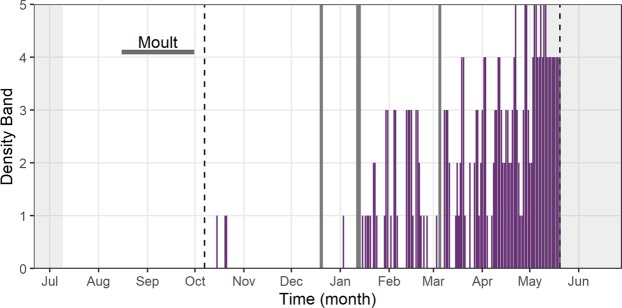
Population level colony attendance of common guillemots during the non-breeding season. Population level non-breeding season colony attendance of a study plot of guillemots at the Isle of May, derived from camera data. Periods of no data during the non-breeding season are shaded in dark grey. The breeding season shaded in light grey and the horizontal dark bar corresponds to the moult period. Vertical dashed lines indicate the start and end dates of the camera data respectively.

Inspection of time-lapse camera data revealed that guillemots first returned to the colony after the breeding season on the 15^th^ October but colony attendance remained low until early January (Fig. 3). Throughout the winter, low numbers of guillemots attended the colony during the early morning, with birds typically arriving just before dawn. The length of time that guillemots were ashore varied greatly from <1 hour to all day, but during November – January occupancy was usually <4 hours. From late January onwards there was a gradual increase in the proportion of guillemots that were at the colony (Fig. 3). Thus, by early April some birds were ashore every day and an increasing proportion of sites were occupied for a greater proportion of the day (Fig. 3). There was a continuation of this pattern into the breeding season, with birds found increasingly at the colony (Fig. 3).

**Figure 4:**
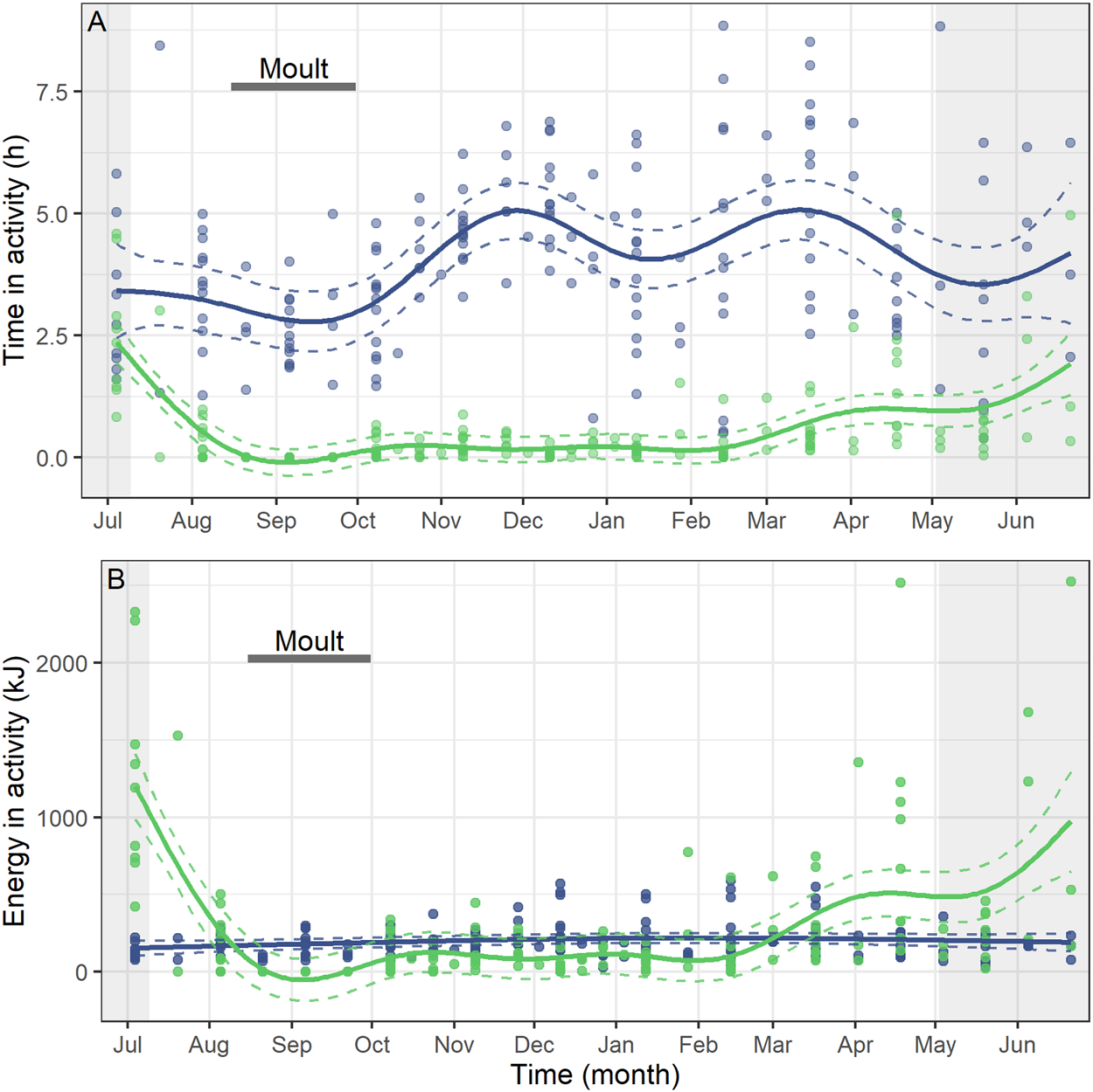
Annual daily time spent diving and flying by common guillemots and the daily energetic cost of these activities. (**A**) The daily time spent a) diving (blue) and b) flying (green) by common guillemots from the Isle of May. (**B**) The daily energetic costs of a) diving (blue) and b) flying (green). For both panels, data span the annual cycle (4^th^ July 2005–21^st^ June 2006). Mean estimated smoothing function (solid line) with upper and lower confidence intervals at two standard errors above and below the mean (dashed lines) from generalized additive mixed models. We also present the raw data points. The breeding season is shaded in grey and the horizontal dark bar corresponds to the moult period.

Data from the activity loggers indicated that the time that guillemots spent diving and flying each day varied over the annual cycle, with no substantial effect of sex (Supplementary Table S1). During the period of the year that they were volant, the total time that guillemots spent flying per day was also generally low (Fig. 4A), particularly between October and February (0.2 ± 0.03 hours). During this winter period, flight time was less than 0.5 hours for 90% of days and the maximum daily flying time was 1.52 hours for an individual on 28^th^ January. The longest individual flight that we identified during this winter period was 0.7 hours on 13^th^ February. Between March and June during pre-breeding, incubation and chick rearing, the GAMM predicted a gradual increase in average daily flight time from 0.72 ± 0.03 to 1.58 ± 0.07 hours (Fig. 4A). This resulted in a marked increase in flight-related energy expenditure and thus contributed to the rise in DEE across the non-breeding period (Fig. 1), although there was no substantial effect of sex (Supplementary Table S1, Fig. 4B).

Guillemots spent considerably more time diving than flying throughout the annual cycle (mean dive time per day = 4.10 ± 0.13 hours; Fig. 4A). Time spent diving was high during the breeding season (May, June and July mean = 3.68 ± 0.43 hours), but was even higher during the non-breeding period, particularly in November – December (4.85 ± 0.18 hours) and in March (5.62 ± 0.43 hours). Contrastingly, the GAMM predicted a minimum time spent diving per day of 2.78 hours on 12^th^ September. Despite large temporal fluctuations in daily dive time (Fig. 4A), as the energetic costs of diving were relatively low, this did not translate into large variation in the daily energetic cost of diving across the annual cycle, although this contribution was slightly higher in December – March than during the rest of the year (Supplementary Table S1; Fig. 4B).

**Figure 5:**
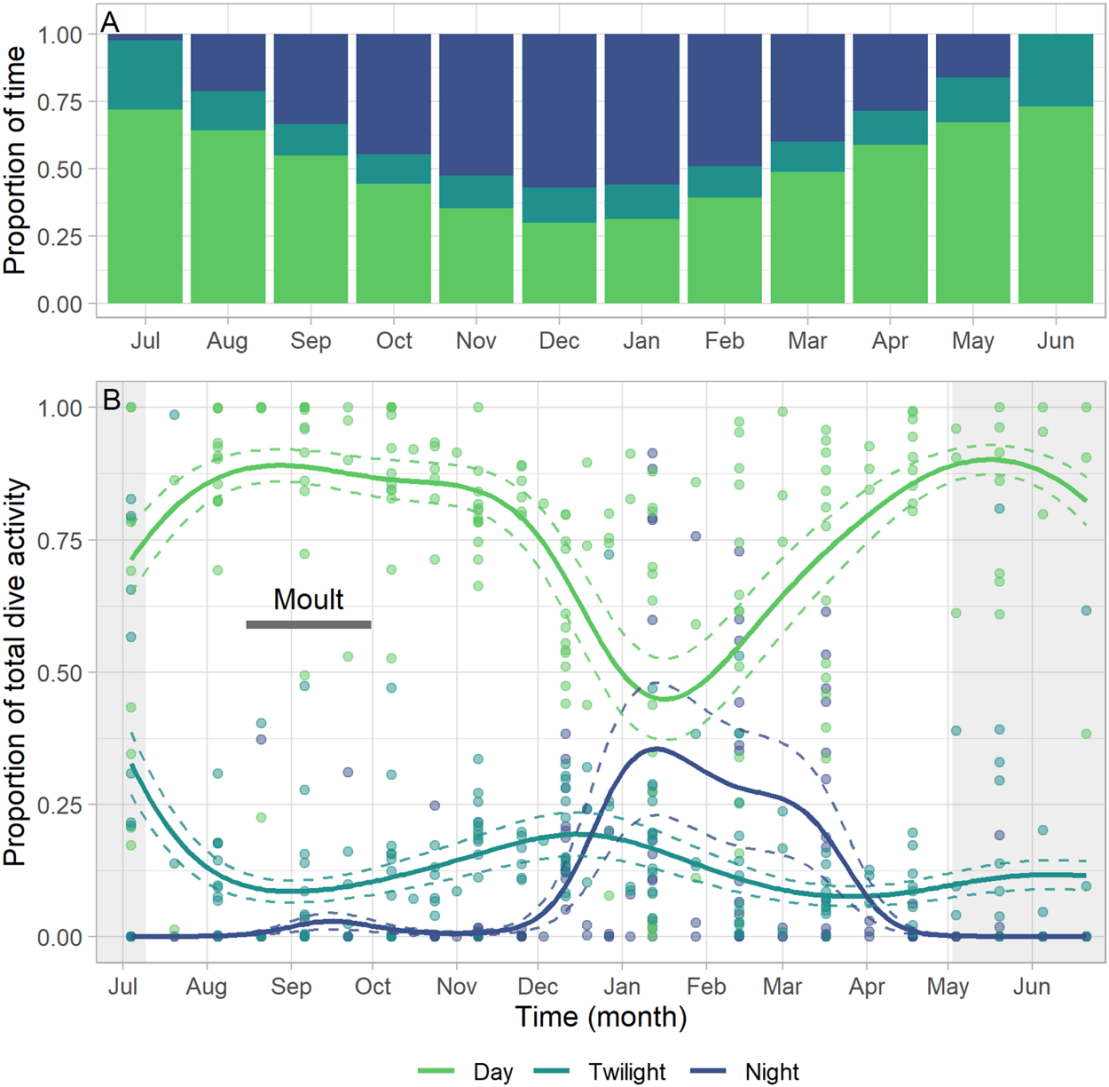
Annual proportions of day, twilight and night and the proportion of diving activity that occurred during these conditions. (**A**) The proportion of the 24 hour period made up of day, twilight and night for each month, averaged across the individuals’ locations and hence environmental conditions. (**B**) The proportion of total dive activity that took place during day, night and twilight throughout the annual cycle (4^th^ July 2005–21^st^ June 2006). Mean estimated smoothing function (solid line) with upper and lower confidence intervals at two standard errors above and below the mean (dashed lines) from generalized additive mixed models. Data are from common guillemots from the Isle of May. We also present the raw data points. The breeding season is shaded in grey and the horizontal dark bar corresponds to the moult period.

Due to the temperate location of the guillemots, light availability varied greatly throughout the annual cycle with the proportion of daylight decreasing throughout the winter, corresponding with an increase in nocturnal conditions (Fig. 5A). During June and July, higher proportions of the 24 hour period were subject to twilight as opposed to night than the rest of the year (Fig. 5A). Throughout the year, the majority of dives occurred during daylight hours (proportion = 0.74 ± 0.02; Fig. 5B). However, the proportion of total dive activity that occurred during daylight did vary temporally (Supplementary Table S1) and was lowest in December – February (proportion = 0.57 ± 0.04). Nocturnal diving activity increased at this time (Fig. 4B; mean annual night diving activity = 0.46 hours ± 0.08; December – February diving activity = 1.18 hours ± 0.23) and did not always coincide with a reduction in the total hours of available daylight (Fig. 5A). The proportion of diving activity occurring during twilight also varied (Supplementary Table S1), and was highest in July, when twilight composed a higher proportion of the 24 hour period, but not in June, when the daily proportion of twilight conditions was also high (Fig. 5A). Sex did not have a substantial effect on the proportion of time spent diving during different daylight conditions (Supplementary Table S1).

**Figure 6:**
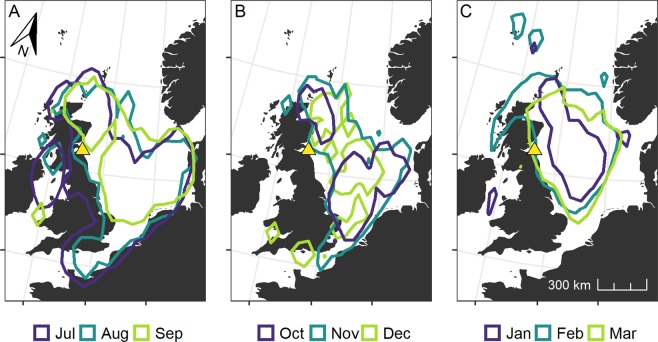
Core areas utilised by common guillemots from the Isle of May outside the breeding season. Core use areas (50% kernel density contours) of 13 common guillemots from the Isle of May (location illustrated with a yellow triangle) according to month and period of the non-breeding season. Map prepared with R packages *ggplot2*^71^*, ggspatial*^72^*, gridExtra*^73^ and *rworldmap*^74^.

Following the breeding season, guillemots moved away from the Isle of May and migrated to areas within the North Sea. Some individuals reached the English Channel and the Irish Sea (Fig. 6A). Initially, after departing the breeding colony in July and August, as DEE began to decrease (Fig. 1), guillemots were widely distributed across the North Sea (kernel home ranges: 624,682 and 477,953 km^2^ respectively). Distribution was more restricted during December (Fig. 6B) and January (Fig. 6C; kernel home ranges: 163,436 and 158,863 km^2^ respectively) when the majority of the tracked population was concentrated within the western and northern North Sea. However, from January onwards, during the period of increasing DEE, guillemots moved progressively closer to the Isle of May (Fig. 6C), still using the central part of the northern North Sea. These movements are consistent with the evidence that they were spending an increasing amount of time at the colony (Fig. 2), necessitating increasing flight time between the feeding areas and the breeding sites (Fig. 4A).

## Discussion

Animals must adapt their behaviour and energetic budgets in order to survive the seasonally varying environmental conditions that they face during their annual cycles. However, due to their challenging nature, the number of studies that have sought to investigate the behavioural and energetic strategies that free-ranging animals utilise to survive annual cycles is relatively small. We show that in this population, surviving guillemots managed their energy expenditure throughout the year by adjusting their activity budgets and demonstrating behavioural plasticity by foraging nocturnally. Daily Energy Expenditure (DEE) during the breeding season was largely higher than during the non-breeding period (Fig. 1) due to thermoregulatory costs and increased flight activity associated with colony attendance. However, despite heightened energetic costs, values of DEE remained below the proposed maximum sustainable threshold (4 to 5 × BMR^6,17^), even at its peak (GAMM prediction of DEE during April = 2212 kJ; 3.8 × 580 kJ BMR^23^).

Thermoregulatory costs can form large components of animal energy budgets^24^, particularly those of seabirds that spend large proportions of time within the marine environment, where thermoregulation provides a heightened energetic challenge^25^. The DEE equation (Eq. 1) that we utilised incorporated the seasonally varying thermodynamic costs of different values of SST during the periods that birds spent both active and inactive on water. We therefore observed a year-round pattern in DEE that partially mirrored that of SST (Fig. 2). High values of SST corresponded to the main moult period when guillemots were flightless and both dive activity and DEE were low. During the moult period, kernel analyses indicated that guillemots were widely distributed throughout the North Sea (Fig. 6A). This pattern of migration was likely to be representative of individuals targeting productive areas, perhaps within multi-species flocks, where they could remain without making major flights^26,27^. Utilising productive areas, particularly during times of higher SST which may in turn affect prey availability and quality, might therefore allow the repartitioning of energetic resources to the intrinsically costly process of feather renewal^28^ and allow the accumulation of fat reserves^29^. Indeed, it is likely that our values of DEE during the moult period are underestimates as they do not account for the intrinsic cost of moult^30^. For many migratory birds, primary feather moult takes place shortly after the breeding period^31,32^. The post-reproductive timing of moult might be particularly crucial for guillemots which need to replace feathers that have been physically abraded and in contact with guano whilst they were breeding on densely populated cliff ledges^33^. Additionally, the occurrence of moult prior to a decrease in SST (Fig. 2) could also be advantageous in terms of acquiring high-condition plumage and subsequent decreased thermoregulatory costs^25,34^.

Following the moult period, guillemot DEE gradually increased across the subsequent winter months before reaching a non-breeding season peak in April, followed by similarly high values during the breeding season (Fig. 1). Seabird energy expenditure is high during reproduction and generally increases across the breeding period^35^. Here, increasing values of guillemot energy expenditure throughout the non-breeding period were largely driven by heightened flight activity (Fig. 4), associated with increased colony attendance (Fig. 3). Whilst they are well adapted for wing-propelled diving, flight is an energetically costly activity for guillemot species, with Brünnich’s guillemots *U. lomvia* having the highest flight costs recorded for any vertebrate^14^. Contrastingly, in albatrosses flight is highly efficient because they are able to use winds to soar and glide, as opposed to engaging in the costly flapping flight utilised by auks^36^. Flight activity can therefore form high proportions of albatross time-activity budgets (e.g. 12.7 hours per day in incubating black‐browed albatrosses *Thalassarche melanophrys*) and yet result in relatively low values of mass-specific DEE^37^. Guillemots, however, must adapt their behaviour and reduce their time spent flying in order to minimise its contribution to DEE. Guillemots from the Isle of May did reduce their flight activity from October to March, in comparison to the breeding season; it seems possible that the guillemots located productive North Sea foraging areas and stayed within these, likely making short commuting flights to optimal feeding patches, as opposed to extensive daily movements^38^. However, as the breeding season neared and birds became constrained to remaining in proximity to the colony to occupy and defend their breeding sites^39^, flight activity and the associated energetic costs increased (Fig. 4), leading to heightened DEE (Fig. 1). Indeed, guillemots are under central-place foraging constraints during the pre-breeding and breeding periods, undertaking longer flights than during the winter, commuting to and from the colony on a daily or near-daily basis, with detrimental consequences for their energy expenditure. Contrastingly, energetically advantageous lower thermoregulatory costs may be likely whilst birds are at the colony as they will be exposed to warmer air temperatures, lower conductivity and increased body heat conservation through sharing warmth with conspecifics^40^. Overall, Isle of May guillemots chose to return to the colony outside the breeding season, indicating that despite its associated energetic costs, this must have been a beneficial strategy that local resources were able to support.

Coinciding with high values of DEE during the pre-breeding period (Fig. 1), guillemot dive activity showed a peak during this time (March), as well as during November – December (Fig. 4A). These two non-breeding season peaks in dive activity may be due to increased foraging effort: the first peak a response to high thermoregulatory costs and winter storm events^41,42^ and the second due to the nutritional requirements associated with attaining pre-breeding condition. Although, like penguins, guillemots are generally well adapted for diving, their efficiency at locating and capturing prey may be hindered by stormy conditions causing fish shoals to disperse and the daily vertical migrations of prey to be disrupted^41^. Additionally, guillemots have longer wings and lighter body masses than penguins, which leads to increased drag, differences in buoyancy levels and dive costs being 30% higher than they would be in a similar-sized penguin^14^. These peaks in guillemot dive activity were therefore associated with a small increase in energetic costs (Fig. 4B) and DEE^43^.

Had we observed an overlap in the timings of peak dive effort and peak nocturnal diving, this would have indicated that guillemots were under constraints and had to feed at night. Instead, there was a mismatch in the timings of two peaks in dive activity and a peak in nocturnal diving, which was highest during January – March (Fig. 5B). This mismatch therefore suggests that guillemot foraging was not constrained by shorter days and instead nocturnal diving may have been an active choice. Guillemots may have been influenced by moonlight availability whereby birds diversified from foraging largely on lesser sandeels (*Ammodytes marinus*) during the breeding season, to consuming nocturnally-available prey resources, such as bottom-dwelling and mid-water fish species, during the winter^44,45^. By adopting a seasonally optimal foraging strategy that tracks the availability of prey resources, guillemots may benefit from increased self-provisioning opportunities and the maintenance of high body condition throughout the non-breeding period^46^.

Despite our values of DEE being based on a number of assumptions^21^, we are confident in the patterns of guillemot behaviour and energetic expenditure that we have identified for this annual cycle. Although the pattern of DEE is different to those that have been recorded in guillemot populations breeding in Newfoundland, Canada^21^ and Svalbard, Norway^20^, our estimates are within the ranges reported within these studies. For each of these high latitude guillemot populations, the winter (January – February) was an energetically challenging period. Whilst we also found a gradual increase in energy expenditure throughout the late winter, we suggest that Isle of May guillemots adapted their behaviour in order to balance their energy budgets and maintain a reasonable level of DEE throughout the year, which also allowed them to return to the colony during the pre-breeding period. Indeed, our study provides estimates of guillemot DEE for a full year for the first time, hence enabling us to investigate the strategies utilised by birds that survived the entire annual cycle during a winter when the return rate of adult guillemots and their subsequent breeding success was extremely low^22^. Furthermore, we describe a previously unidentified non-breeding season peak in DEE during the pre-breeding period, driven by low values of SST, central-place foraging constraints and high associated costs. We hypothesise that despite this pre-breeding peak in DEE and high values throughout the breeding period, surviving guillemots displayed adaptive measures to manage their energy budgets, under localised temperate conditions, and keep them below an energetically sustainable threshold. We therefore provide evidence to support the hypotheses that guillemots exhibit behavioural plasticity, identifying nocturnal foraging, non-breeding colony attendance and modulated flying and diving effort as responses to seasonally varying environmental conditions. By using guillemots as a model through which to investigate year-round responses to seasonal environmental conditions, we emphasise the importance of understanding the interplay between energetic constraints and behavioural strategies and their links to survival and population dynamics.

## Materials and Methods

### Data collection

Fieldwork took place on the Isle of May National Nature Reserve, Scotland (56° 11′N, 02°33′W) from 2005–2006. The mean population fledging date (10^th^ July 2005) and the first guillemot egg date (2^nd^ May 2006) were obtained from long-term monitoring plots at the colony, using standardised methods^47^.

During June 2005, we captured 30 adult guillemots that were brooding young chicks. We attached global location sensing (GLS) time depth recorder (TDR) devices (LT2400, Lotek Wireless, St John’s, Newfoundland, Canada, 36 × 11 mm) to Darvic leg-rings under British Trust for Ornithology and Scottish Natural Heritage licences (licence numbers C/4671 and 5632 respectively). Device plus ring mass (6.5 g) was 0.69% of the birds’ mean breeding body mass at recapture. During the 2006 breeding season, 13 adult guillemots were recaptured (43% retrieval rate; pre-breeding n = 3 and early chick-rearing n = 10) and the loggers were removed. We therefore obtained data from birds that survived the annual cycle. Three to five body feathers were collected from recaptured birds under UK Home Office Licence to enable birds to be sexed using two CHD I genes^48^. All procedures were conducted in accordance with relevant UK guidelines and regulations and were approved under research licences issued by Scottish Natural Heritage.

Individuals from the Isle of May population are known to return to the colony after the autumn flightless period and during the winter, although they do not attend the colony during the night in the nonbreeding period^49^. We quantified population-level daytime non-breeding season colony attendance behaviour using daily time-lapse photography from 7^th^ October 2005 to 20^th^ May 2006. A camera was trained on a sub-colony of guillemots approximately 50 m north of the instrumented birds. We assigned a daily density score of between zero and five, based on the number of individuals at the study plot (0 = no individuals; 1 = <10 individuals; 2 = 10–20 individuals; 3 = 20–50 individuals; 4 = > 50 individuals; 5 = all ca. 100 breeding sites occupied).

### Spatial data

Loggers employed internal processing algorithms to calculate daily location fixes; latitude and longitude were calculated on-board the devices based on estimates of day length and the timing of midday^50^. Locations were re-estimated using an iterative forward step selection framework through the *probGLS* package^51^. Improved location estimates were generated by calculating a cloud of possible locations (n = 1000) and weighting these according to 0.25° resolution NOAA optimally-interpolated sea surface temperature and daily median SST recorded by the logger^52–54^. Based on these weightings, 100 likely movement paths were computed and the geographic median for each location cloud was then selected. This method allowed estimations of locations around the equinoxes to be computed^54^. Despite the improved estimates generated using this algorithm, 6 days prior to each equinox were removed following visual inspection (locations between the 10^th^ – 15^th^ September 2005 and 11^th^ – 16^th^ March 2006). Next a cost-path analysis was conducted and fixes that indicated movements of over 750 km in 12 hours were removed (0.6% of all fixes; cut-off assigned based on a maximum flight speed of 60–70 kmh^−1^ ^55^). These fixes typically resulted from light interference at dawn and dusk, perhaps caused by the logger being shaded e.g. if the leg was tucked into the feathers. Erroneous fixes were also frequent when the birds were thought to be at the breeding colony. Therefore, fixes that occurred prior to the 2005 mean fledging date and those that occurred after the date of the first guillemot egg in 2006 were assigned Isle of May coordinates.

Population-level monthly kernel home ranges for the non-breeding period which was taken to be from 10 days after the mean fledging date to 30 days before the first egg date (i.e. 20^th^ July 2005–2^nd^ April 2006) were created using the *adehabitatHR* package^56^, using a least squared cross validation method and a 50 km grid size. Core use areas during the non-breeding period were represented by 50% kernel density contours^21^.

Daily air temperature was extracted from either Lerwick (60° 14′N, 01°18′W), Leuchars (56° 38′N, 02°86′W), Bridlington (54° 1′N, 00°21′W) or Sandettie (51° 10′N, 01°80′W) weather stations^57^, depending on which was in closest proximity to the centroid of an individual’s fortnightly GLS fix cluster. These individual-specific fortnightly locations were also used in conjunction with the *maptools* package^58^ to calculate location-specific times of sunrise, sunset and nautical twilight (when the sun was between 6 and 12 degrees below the horizon) for each day for each bird.

### Activity data

Loggers recorded time, depth (to the nearest 0.01 m) and temperature (to the nearest 0.01 ^o^C). Due to the limited memory size of the loggers and to record data over an extended period of the annual cycle, loggers recorded a depth and temperature reading either every 16 s for a 24 h period every 30 days (n = 9) or every 32 s for a 24 h period every 15 days (n = 4)^13^. Dive and temperature data were available for all birds until March (n = 13), for 10 birds until April, 9 until May and 3 until June. The dive data were corrected for device drift using the *diveMove* package^59^.

Days which did not have temperature and depth data for the entire 24 hour period were removed (n = 9) and the remaining data (n _bird days_ = 179) were utilised to derive daily time-activity budgets. Time-activity budgets were based on the identification of five key activities: a. diving (${T}_{d}$), b. flying (${T}_{f}$), c. at the colony (${T}_{c}$), d. active on water (${T}_{a}$) and e. inactive on water (${T}_{i}$)^21,60^. Active on water (${T}_{a}$) included intervals on the surface between dives and longer periods between dive bouts when activities such as swimming and preening were undertaken. Inactive on water (${T}_{i}$) was taken to represent resting time when guillemots withdrew their leg and foot into their plumage^60^.

Our behavioural classifications were based on a set of decisions appropriate to the resolution of the data at our disposal. Supplementary Fig. S2 gives examples using this behavioural classification for representative periods of the annual cycle. Identification of different behaviours was made sequentially. First, times when loggers recorded depths of >1 m, were assigned as diving (${T}_{d}$). We summed the time spent diving each day to give the daily time spent diving. We also calculated the proportion of time spent diving during daylight, night or nautical twilight during each 24 hour period, based on the location-specific times of sunrise, sunset and twilight. We extracted logger temperatures during these dives (T) to estimate the range of water temperature values that guillemots encountered whilst foraging on that day. Next, we classified all non-diving behaviours. We inferred that when the temperature recorded by the logger was greater than T – 0.2 ^o^C and less than the 75% quartile of T + 1 ^o^C, birds were active on the water (${T}_{a}$)^61^.

We then split the annual cycle into different periods, based on the population-level phenology data, to further refine time-activity budgets based on a priori knowledge of sex- and period-specific drivers of guillemot activity in this particular population. As the mean population-level fledging date in 2005 was the 10^th^ July, we assumed that instrumented birds could have been at the colony (${T}_{c}$) until the 20^th^ July and thus could have been engaged in any of the five behaviours during this time (Supplementary Fig. S2A). Bouts of flight (${T}_{f}$) were separated from bouts of inactivity (${T}_{i}$) on the assumption that guillemots do not fly at night^61,62^ and logger temperature being less than daily air temperature + 4 ^o^C, as opposed to the higher temperatures expected during leg-tucking events (${T}_{i}$^21,60^). We validated this classification of ${T}_{f}$ and ${T}_{i}$ by modelling the impact of behavioural state on the relationship between the duration of time spent in the activity and the maximum temperature recorded (Supplementary Fig. S3). ${T}_{f}$ was summed per day in order to calculate the daily time spent flying.

From 20^th^ July until the assumed start of primary feather moult (15^th^ August), female birds may have engaged in flight, but male birds were assumed to be at sea with their chicks and not to fly. We further assumed that all birds underwent primary feather moult between mid-August (15^th^) and the end of September (30^th^) during which time they were flightless. We therefore forced the data into three known activities during these periods (${T}_{d}$, ${T}_{a}$ or $\,{T}_{i}$; Supplementary Fig. S2B).

Based on previous studies, guillemots were assumed to be absent from the colony (no ${T}_{c}$) between the end of the breeding season (20^th^ July) and November 15^th^. Previous studies of individually marked birds have not detected any difference between the sexes in return dates^63^. Thus both males and females were assumed to be absent from the colony during this time and data were forced into the four other activities (excluding ${T}_{c}$) during this period. From the 2^nd^ April (30 days prior to the date of the first guillemot egg in 2006) guillemots were assumed to be able to spend an increasing amount of time at the colony, based on previous intensive visual observations^64^. During this period we identified ${T}_{c}$ activity as times when temperature recorded by the logger was less than the daily median recorded during leg-tucking events (${T}_{i}$; Supplementary Fig. S2C). Outside this period, when the logger temperature was elevated for over half an hour during the morning, we identified ${T}_{c}$ activity as before. We classified periods of increasing logger temperature that occurred immediately before ${T}_{c}$ events as $\,{T}_{f}$, and periods immediately after ${T}_{c}$ as $\,{T}_{a}$, as birds fly to the colony before periods ashore and later land on the water immediately after departing from the colony (as also observed in Brünnich’s guillemots^60^).

### Daily energy budgets

We combined daily time-activity budgets with estimates of activity-specific energy costs to determine the daily energy expenditure (DEE in kJ) utilising an equation based on Brünnich’s guillemots^14,21,61^:

1 $$DEE=508\,{T}_{f}+33\,{T}_{c}+1.01{\sum }^{}[1-\,{e}^{\frac{-Td}{1.23}}]+(113-2.75\,SST){T}_{a}+(72.2-2.75\,SST){T}_{i}$$

where ${T}_{d}$ represents individual dive durations in minutes (which were then summed for each day) and ${T}_{f}$, ${T}_{c}$, ${T}_{a}$ and ${T}_{i}$ represent hours in flight, at the colony, active on water and inactive on water respectively as previously defined. SST was the fortnightly mean recorded by the logger. We also calculated the daily energetic costs of flight and diving separately based on their relative contributions to Eq. 1.

### Data analyses

Data exploration indicated potentially non-linear relationships in temporal patterns of guillemot DEE, SST, activity, activity costs and diving behaviour throughout the annual cycle and we therefore implemented generalised additive mixed models (GAMMs) using the *gamm4* package^65^. A similar approach has been adopted in studies of activity budgets of overwintering Laysan albatrosses *Phoebastria immutabilis* and black-footed albatrosses *P. immutabilis*^66^, and migrating Cory’s shearwaters *Calonectris borealis*^67^. We included day since logger deployment (‘dDay’) as a smoothing function because of its many levels, and sex as a fixed categorical factor. Individual bird was modelled as a random effect, with random intercepts, to account for the dependency structure present in the data^68^. We used a Gaussian distribution with an identity link function for GAMMs with the response variables of DEE, SST, daily time spent in flight, daily time spent diving, daily energetic cost of flight and daily energetic cost of diving (although sex was not included within the SST model). To model the proportion of total dive activity (daily time spent diving) that took place during daylight, night or nautical twilight we used a binomial distribution with a logit link function within three GAMMs. The predictor variables and random effects used within these binomial models were the same as those used in the previously described set of GAMMs. We validated the models by plotting the residuals against the fitted values and the model covariates^68^.

All analyses and plotting were conducted utilising R version 3.2.3^69^; all values are means ± standard error and all times are UTC.

## Supplementary information

Supplementary information.

## Data Availability

All data will be made available from the Environmental Information Data Centre: 10.5285/bd24da1f-0761-4564-8dd8-dfd71a559a71 (Dunn *et al*. 2020)^70^.
