# Supplementary material for: A year in the life of a North Atlantic seabird: behavioural and energetic adjustments during the annual cycle
Source: Sci Rep. 2020 Apr 7;10:5993. doi: 10.1038/s41598-020-62842-x (PMC7138806; doi:10.1038/s41598-020-62842-x)
Supplement: Supplementary file 1 — Supplementary information. [file 41598_2020_62842_MOESM1_ESM.docx]

# A year in the life of a North Atlantic seabird: behavioural and energetic adjustments during the annual cycle

Ruth E Dunn, Sarah Wanless, Francis Daunt, Mike P Harris, Jonathan A Green

Supplementary information

### Supplementary Table S1

**Table S1|** Results of generalised additive mixed models (GAMMs). Significant P-values are highlighted in bold.

| Response | Model term | *df* | *F* | *P-value* |
| --- | --- | --- | --- | --- |
| DEE | s(dDay) | 6.64 | 14.87 | **<0.001** |
|  | Sex | 2 | 0.22 | 0.81 |
| SST | s(dDay) | 8.06 | 311.5 | **<0.001** |
| Time spent diving | s(dDay) | 6.92 | 6.00 | **<0.001** |
|  | Sex | 2 | 0.62 | 0.54 |
| Time spent flying | s(dDay) | 7.54 | 18.39 | **<0.001** |
|  | Sex | 2 | 0.75 | 0.48 |
| Energetic cost of diving | s(dDay) | 2.12 | 3.07 | **0.04** |
|  | Sex | 2 | 0.18 | 0.83 |
| Energetic cost of flying | s(dDay) | 7.54 | 18.39 | **<0.001** |
|  | Sex | 2 | 0.75 | 0.48 |
| Response | Model term | *df* | *χ^2^* | *P-value* |
| Diving activity occurring during daylight | s(dDay) | 8.99 | 223521 | **<0.001** |
|  | Sex | 2 | 6.57 | 0.06 |
| Diving activity occurring during twilight | s(dDay) | 8.99 | 56996 | **<0.001** |
|  | Sex | 2 | 5.20 | 0.07 |
| Diving activity occurring during night | s(dDay) | 8.99 | 197079 | **<0.001** |
|  | Sex | 2 | 1.81 | 0.40 |

### Supplementary Figure S2


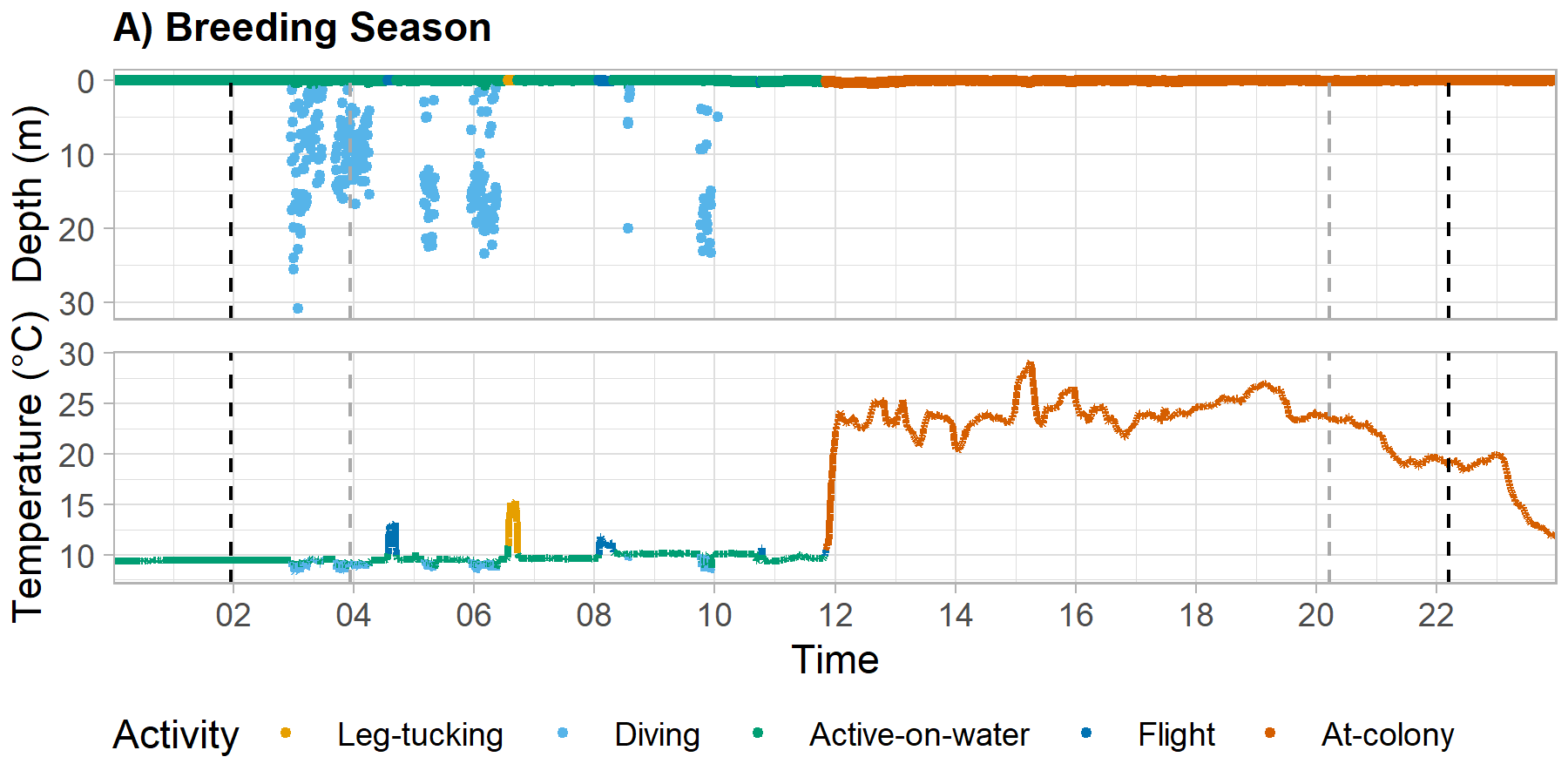


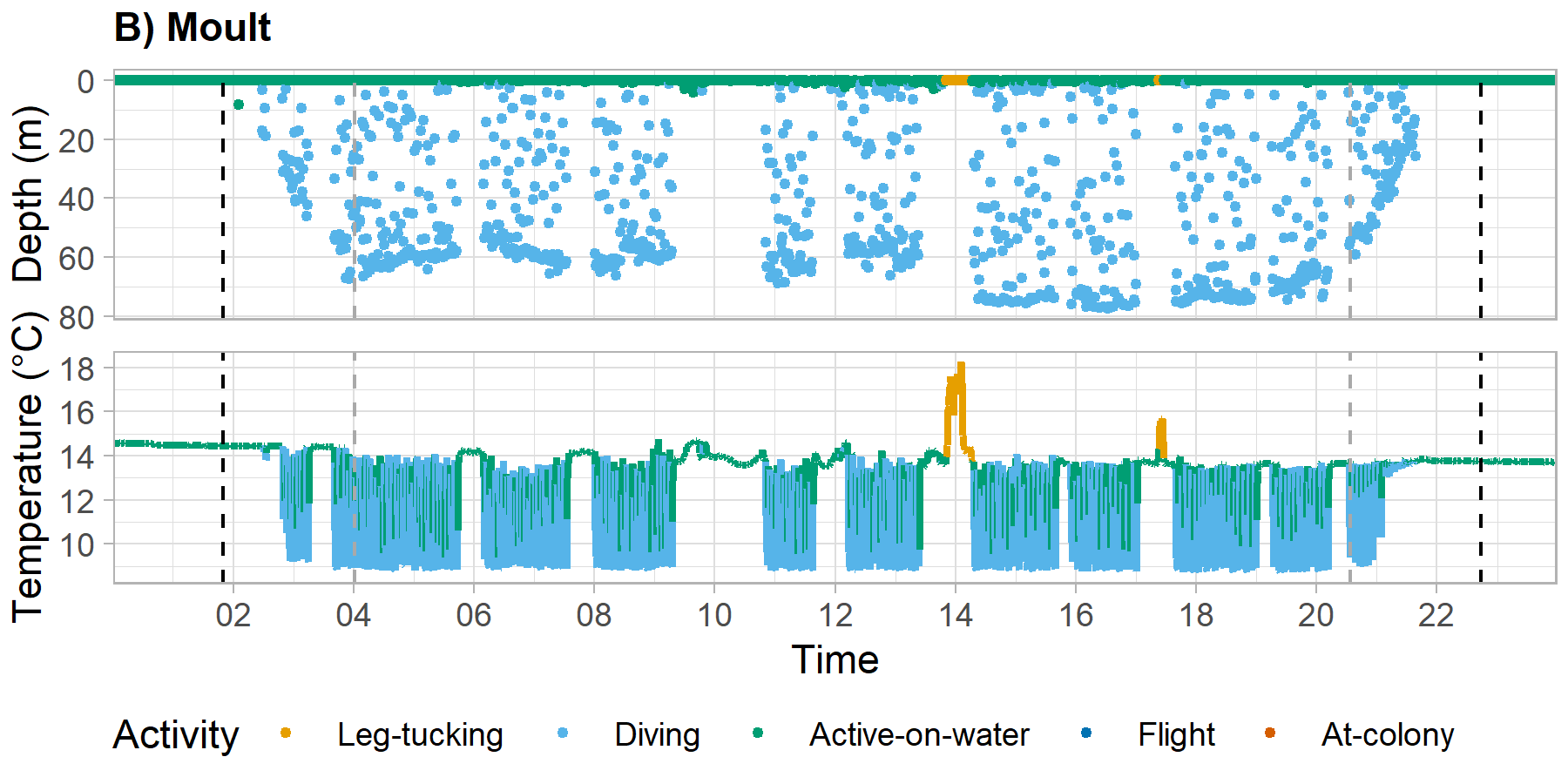


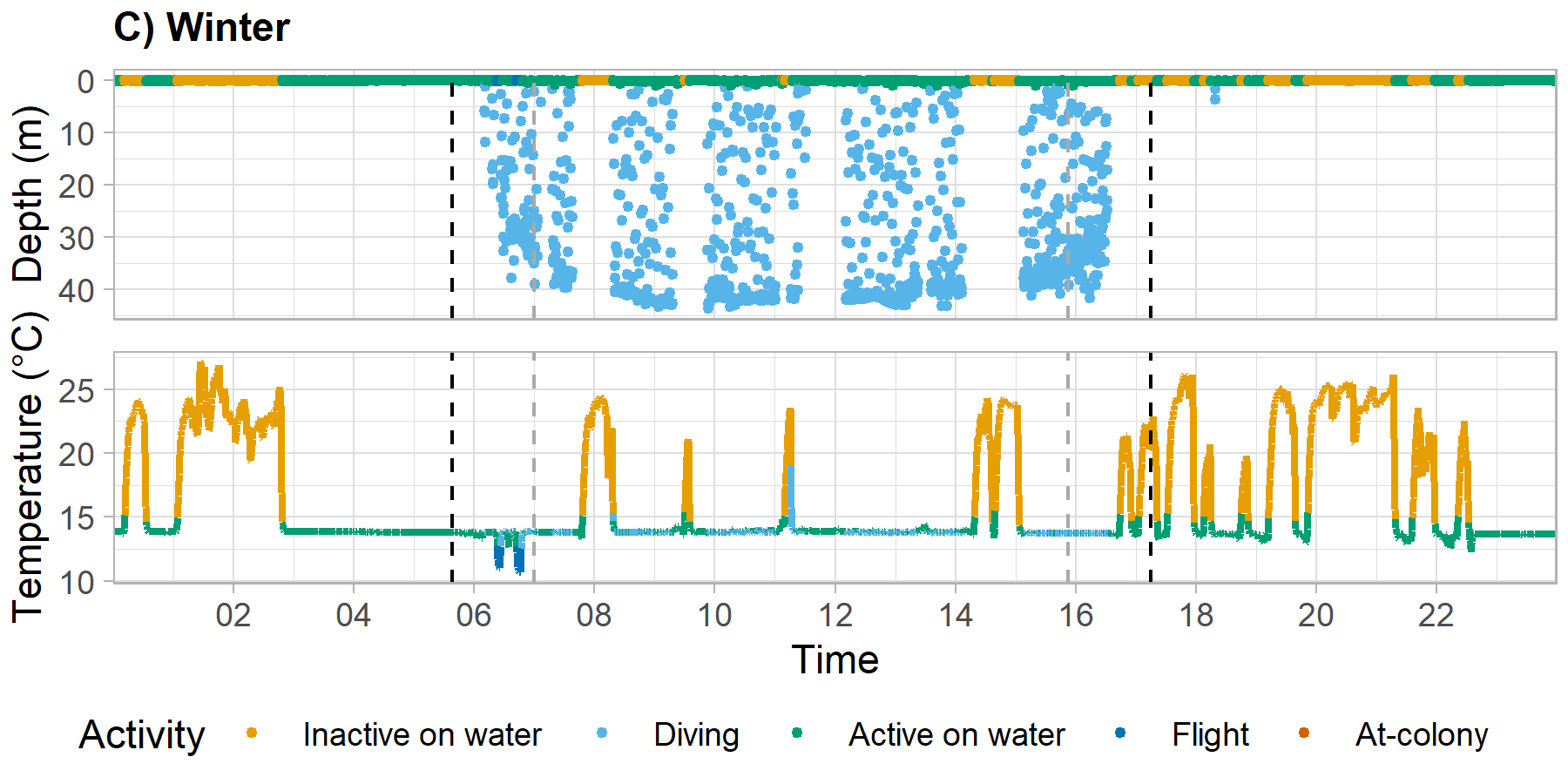


**Figure S2|** Examples of results of the classification of guillemot activity from depth and temperature profiles during three key periods of the annual cycle. Grey dashed vertical lines indicate the start of day and dusk respectively; Black dashed vertical lines indicate the start of dawn and night respectively.

### Supplementary Figure S3

We assessed the classification of logger temperature data into flight behaviour ($T_{f}$) and inactive on water ($T_{i}$) by fitting a mixed model using the lmer function in the lme4 package (Bates et al. 2015). The interaction between log-transformed time-in-activity (a continuous variable) and behavioural classification (a two-level categorical variable) was fitted as a predictor of log-transformed maximum temperature. Individual bird ID was included as a random factor to account for potential non-independence.
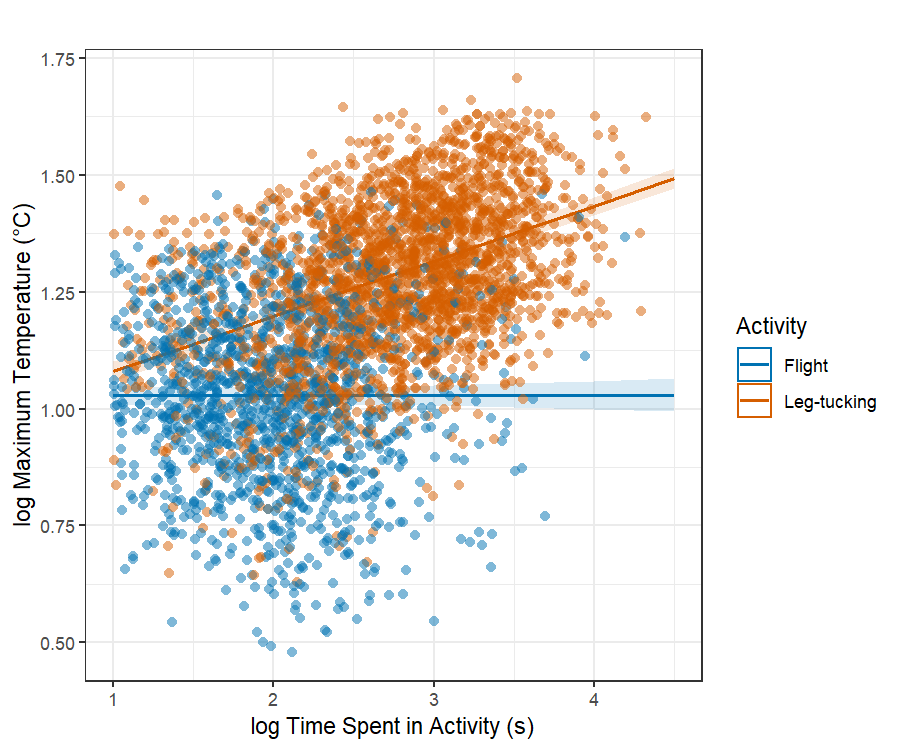


**Figure S3|** The log of the relationship between the amount of time spent in activity (s) and behavioural classification on maximum temperature (^o^C) for common guillemots throughout the annual cycle.

There was a significant interaction between behaviour and bout duration (ANOVA F = 238.97, P < 0.01; Fig. S3), confirming that the nature of the relationship and hence the time taken to reach maximum temperature differed between leg-tucking and flight behaviour. For leg-tucking ($T_{i}$), longer times incurred higher temperatures but with an asymptote due to temperature approaching body surface temperature. For flight ($T_{f}$), there was no clear relationship because air temperature, and hence ∆T, varied substantially between flights, independent of their duration.
